# Keep on Laying Eggs Mama, RNAi My Reproductive Aging Blues Away

**DOI:** 10.1371/journal.pgen.1004808

**Published:** 2014-12-04

**Authors:** Sandeep Kumar, Zuzana Kocsisova, Kerry Kornfeld

**Affiliations:** Department of Developmental Biology, Washington University School of Medicine, St. Louis, Missouri, United States of America

The study of aging has been dominated by the analysis of degenerative changes in somatic tissues that influence life span, since the decline of life support systems is the cause of age-related death. Reproduction, which is a vital goal of all organisms, also displays an age-related decline. However, this aspect of aging has received much less attention, since the consequence is not death but rather an age-related decline in fertility. Whereas a substantial number of genetic and environmental factors have been demonstrated to influence somatic aging and life span, only a few factors are known to influence reproductive aging. In this issue of *PLOS Genetics*, Wang et al. use an innovative reverse genetic approach to investigate reproductive aging [1].

**Figure 1:**
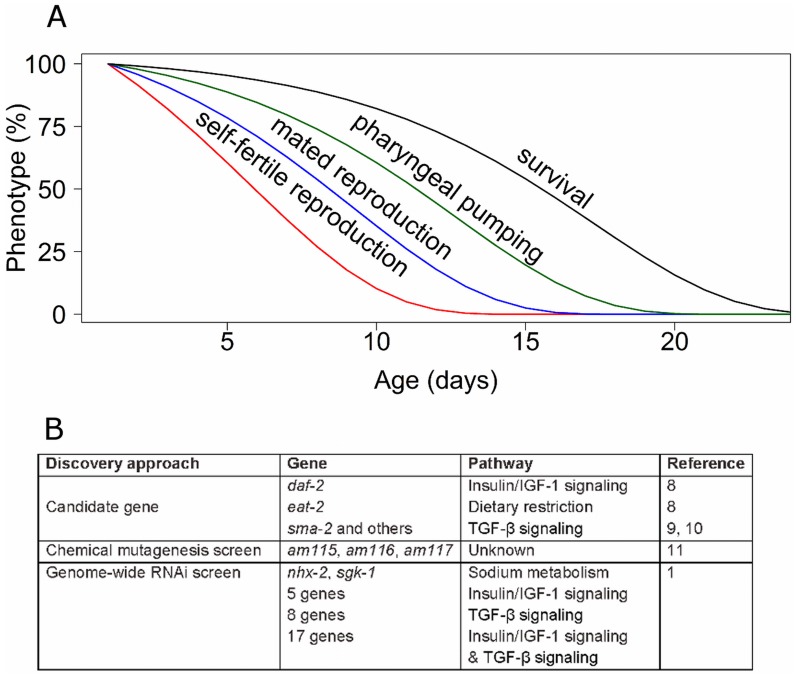
Genetic analysis of reproductive aging in *C. elegans*. (A) *C. elegans* displays a series of age-related degenerative changes including loss of self-fertile reproduction (red), mated reproduction (blue), pharyngeal pumping (green), and survival (black). (B) Three major discovery approaches have been used to identify genes that influence reproductive aging.

Several features of reproductive aging make it an important subject, and the paucity of studies make this a frontier with the promise of new and exciting discoveries. Although reproductive aging is not lethal, it does have important health consequences. In human females, reproductive aging is an important medical issue because the age-related decline in oocyte quality results in increased birth defects and decreased fertility that culminates in reproductive cessation at menopause [2], [3]. Furthermore, reproductive aging is a central issue in understanding the evolution of aging, since it influences progeny number and therefore fitness. The critical questions that are currently being investigated in the area of reproductive aging are (i) what are the genes, pathways, and mechanisms that regulate reproductive aging? (ii) What happens at the molecular, cellular, and tissue level to cause a functioning reproductive organ to degenerate? (iii) What are the relationships between mechanisms that regulate reproductive and somatic aging? Figure 1A shows that the age-related declines of self-fertile and mated reproduction occur well before the age-related declines of neuromuscular activity and survival probability in *Caenorhabditis elegans*. Are mechanisms controlling these declines distinct, overlapping, or identical?

The nematode *C. elegans* is an important model organism for studies of aging [4], [5]. *C. elegans* hermaphrodites have a mated reproductive span of approximately ten days and a postreproductive span of approximately six days for a total adult life span of approximately 16 days. A large number of age-related degenerative changes in reproductive and somatic function have been characterized [6], [7]. Sophisticated genetic approaches have resulted in the identification of many genes that influence somatic aging, demonstrating important roles for insulin or insulin-like growth factor (IGF-1) signaling, mitochondrial function, chemosensory function, and dietary intake in modulating adult life span [5].

Two approaches have previously been used to identify genes that modulate reproductive aging in *C. elegans*: analysis of existing mutations in candidate genes that cause somatic aging delays or other phenotypes, and forward chemical mutagenesis screens (Figure 1B). The candidate gene approach resulted in the discovery that reducing the activity of *daf-2*, which encodes an insulin or IGF-1 receptor, delays reproductive aging [8]. Dietary restriction extends the life span of many animals, and *eat-2* loss-of-function mutations cause dietary restriction by reducing food ingestion. *eat-2* mutants display delayed reproductive aging [8]. Mutations of several genes in the TGF-β Sma or Mab signaling pathway, such as *sma-2*, cause delayed reproductive aging [9], [10]. Thus, high caloric intake and the activity of the insulin or IGF-1 pathway and the TGF-β pathway accelerate reproductive aging. In addition to candidate approaches, the unbiased approach of a forward genetic screen using a chemical mutagen identified three mutants with delayed reproductive aging, although the identity of the affected genes has yet to be reported [11].

The Ruvkun lab has been a pioneer in using whole genome RNAi screens to analyze *C. elegans* biology, and here they describe using this reverse genetic approach to identify genes that influence reproductive aging [1]. The advantage of this approach is that it is relatively comprehensive, in this case a library of 18,413 RNAi clones was analyzed, and the identity of positive genes was known immediately. However, the reduction of gene activity caused by feeding RNAi is partial and variable in different tissues. Wang et al. identified 32 genes that extend the self-fertile reproductive span by at least 20% when RNAi is used to decrease gene activity [1]. To determine how these genes relate to previously described pathways that influence reproductive aging, Wang et al. analyzed interactions with the insulin or IGF-1, TGF-β, and in some cases the *eat-2* caloric restriction pathways [1]. Thirty gene inactivations failed to extend self-fertile reproductive span in the background of altered insulin/IGF-1, TGF-β or both, suggesting that these genes may be involved with these signaling networks. Interestingly, the two gene inactivations that caused the largest reproductive span extensions, *sgk-1* and *nhx-2*, did not interact with the previously identified pathways. The molecular identity of these genes suggests a role in sodium metabolism, which has not previously been implicated in reproductive aging (Figure 1B). Manipulations of both *sgk-1* and *nhx-2* were previously reported to extend life span [12].

To identify the tissue where these genes function, Wang et al. reduced gene activity only in the germline; ten gene inactivations caused extended reproduction, suggesting the site of action is the germline, whereas 22 gene inactivations did not, suggesting the site of action is somatic tissue [1]. Thus, genes that function in both tissues appear to influence reproductive aging. Because self-fertile reproductive span is limited by sperm depletion in *C. elegans* [8], Wang et al. analyzed mated hermaphrodites that are not sperm limited. Nineteen gene inactivations caused extended reproduction in mated hermaphrodites [1]. Intriguingly, several RNAi clones that did not cause the phenotype in mated hermaphrodites did cause the phenotype when both males and mated hermaphrodites were exposed, and some RNAi clones caused the phenotype when only males were exposed. These findings suggest that male mating, which includes physical contact as well as sperm and seminal fluid transfer, may influence reproductive aging in hermaphrodites.

To address the role of these genes in somatic aging, Wang et al. analyzed life span. Five gene inactivations extended mean life span, indicating these genes play a role in both somatic and reproductive aging [1].

The analysis of reproductive aging is at an early stage, and the identification of new genetic regulators by an RNAi screen is a timely addition to the previous knowledge of genetic regulators identified by candidate approaches and chemical mutagenesis screens. It appears that the relationship between reproductive aging and somatic aging is complex, since genetic manipulations have now been identified that affect both processes, only somatic aging, or only reproductive aging. Further work is necessary to rigorously determine if there are indeed separate pathways that modulate degeneration of somatic and reproductive function, or whether selective effects relate to the specific genetic manipulations that have been analyzed. The most important and challenging issue to be addressed in future studies is the mechanism of action of these genes in influencing reproductive aging. This is a significant challenge because there is likely to be a cascade of effects that proceeds from the immediate activity of the gene product to the phenotype of reproductive degeneration. Solving these puzzles will require detailed analysis of phenotypes at the molecular, cellular, and organ levels. This also remains the major challenge for the genetic analysis of somatic aging, since comprehensive explanations of how gene activities influence degenerative change in somatic tissues remain elusive. By identifying a new group of genes that influence reproductive aging, Wang et al. establish a critical foundation for mechanistic studies that will elucidate how and why gene activities accelerate the decline of reproduction [1].
